# Confidence in antimicrobial stewardship and infection prevention among French medical residents: a national cross-sectional survey

**DOI:** 10.1093/jacamr/dlag117

**Published:** 2026-06-17

**Authors:** Ibtissam Ait Azzi, Aude Allemang-Trivalle, Noémie Mercier, Charles-Emmanuel Barthelemy, Sandra Fournier, Benjamin Davido

**Affiliations:** Mission Ministérielle de la Prévention des Infections et de L’antibiorésistance, Direction Générale de la Santé, 13 Avenue Duquesne, 75007 Paris, France; Clinical Research Unit, Direction of Clinical Research, AP-HP Université Paris Saclay, Le Kremlin-Bicêtre, France; Mission Ministérielle de la Prévention des Infections et de L’antibiorésistance, Direction Générale de la Santé, 13 Avenue Duquesne, 75007 Paris, France; Mission Ministérielle de la Prévention des Infections et de L’antibiorésistance, Direction Générale de la Santé, 13 Avenue Duquesne, 75007 Paris, France; Service Prévention du Risque Infectieux, Hôpital Saint-Antoine, 184 Rue du Faubourg Saint-Antoine, 75012 Paris, France; Mission Ministérielle de la Prévention des Infections et de L’antibiorésistance, Direction Générale de la Santé, 13 Avenue Duquesne, 75007 Paris, France; Maladies Infectieuses, Hôpital Raymond-Poincaré, Université Paris Saclay, AP-HP, 92380 Garches, France; UMR1173, Université Versailles Saint-Quentin, 78180 Montigny-Le-Bretonneux, France

## Abstract

**Background:**

Antimicrobial resistance (AMR) represents a major global public health threat. Infection prevention and control (IPC) and antimicrobial stewardship are key pillars to limit AMR, yet little is known about medical residents’ perceived preparedness in these areas. The aim of this study was to assess French medical and surgical residents’ self-reported level of confidence (LOC) regarding IPC and appropriate use of antibiotics (AUA) and to explore factors associated with confidence levels.

**Methods:**

We conducted a national cross-sectional self-administered survey among French residents involved in antibiotic prescribing. Confidence levels were assessed using a 0–10 numeric scale across predefined IPC- and AUA-related clinical situations. Demographic characteristics, training setting, exposure to IPC and AUA training modules and awareness of the national AMR strategy were collected. Non-parametric statistical analyses were performed.

**Results:**

A total of 151 residents were included. Overall, confidence ratings were significantly higher for AUA-related situations than for IPC-related situations (*P* < 0.001). Attendance of IPC training modules was associated with higher IPC confidence (*P* = 0.011), whereas attendance of AUA training modules was not significantly associated with AUA confidence. Training in a university hospital showed limited association with confidence levels. Awareness of the national AMR action plan was low, reported by only 17% of participants.

**Conclusions:**

French residents report heterogeneous confidence profiles, with lower perceived confidence in IPC than in antibiotic-related decision-making. These findings support the need for earlier, multidisciplinary and practice-oriented training programmes integrating IPC and antimicrobial stewardship throughout residency.

## Introduction

The current context reveals a continuously increasing prevalence of antimicrobial resistance (AMR) worldwide, including in Europe. Between 2025 and 2050, AMR is estimated to directly cause more than 39 million deaths and to be associated with a broader 169 million deaths worldwide.^1^

During the last decade, according to the European Centre for Disease Prevention and Control (ECDC), France has remained among the five highest consumers of antibiotics in Europe, with 22.1 defined daily doses per 1000 inhabitants per day in 2024.^2^

In 2013, a survey conducted in France showed limited awareness regarding antibiotic prescribing among medical students, with only 27% knowing that more than 80% of antibiotic prescriptions occur in community practice.^3^ These findings support the need for dedicated antimicrobial stewardship (AMS) policies targeting undergraduate and postgraduate medical training, including residency programmes.

In its latest National Action Plan (NAP) on antibiotic resistance in humans (2022–25),^4^ France aimed to reduce the impact of AMR by targeting a 25% reduction in antibiotic consumption between 2019 and 2025. To support this objective, the plan was subsequently extended until 2027. This NAP is structured around two priority pillars for AMR prevention: infection prevention control (IPC) and AMS. In the present study, appropriate use of antibiotics (AUA) was assessed as a practical clinical component of AMS.

In parallel, the French Society for Hospital Hygiene (SF2H) published in 2023 a national core competency framework defining the required skills and knowledge related to IPC for healthcare professionals.^5^

Within this framework, we conducted a national survey among French medical and surgical residents. The primary objective was to assess residents’ self-reported level of confidence (LOC) regarding competencies related to IPC and AUA, assessed as a practical clinical component of AMS.

## Materials and methods

### Settings

We conducted a national, quantitative, self-administered cross-sectional survey among medical and surgical residents.

The questionnaire was developed within the Directorate General of Health by a multidisciplinary team including a pharmacy resident, an infectious disease specialist and a pharmacist. The questionnaire was reviewed by an independent committee composed of a pharmacist and an infection control practitioner (Table S1, available as Supplementary data at *JAC-AMR* Online).

This study was conducted within the framework of the Ministerial Mission for the Prevention of Infections and Antimicrobial Resistance at the Directorate General of Health, over a three-month period from 8 August to 31 October 2024.

### Study participants

All residents practising in France during the study period were eligible.

We included volunteer medical and surgical residents involved in clinical care across all residency training years. Residents not involved in antibiotic prescribing, such as those in anatomopathology or public health, were excluded.

### Data collection and procedure

Residents were recruited through multiple disseminated channels, including emails sent to residents’ associations and professional unions, direct invitations relayed by department heads and social media platforms dedicated to residents.

Two reminders were sent during the inclusion period, using the same communication channels.

Data were collected using a three-part online questionnaire designed and distributed via the Eval&GO^®^ platform. The online questionnaire was accompanied by the following message:

As part of my internship at the Directorate General for Health (Ministerial Mission for the Prevention of Infections and Antimicrobial Resistance), I invite you to complete a three-part questionnaire aimed at assessing your level of confidence regarding two topics: infection prevention and appropriate use of antibiotics. This questionnaire is not intended to evaluate your practices or knowledge, but rather your perception of the adequacy between your initial training and your clinical practice.

An explanatory message was also included in email distributions and social media postings, together with a QR code and a hyperlink to facilitate direct access to the questionnaire.

The questionnaire consisted of three sections. The first section collected demographic and academic characteristics, including previous training, theoretical knowledge sources and self-reported influenza and COVID-19 vaccination status. The second section focused on infection prevention and control (IPC). Finally, the third section focused on AUA.

Only residents involved in antibiotic prescribing were included in the AUA analysis.

In France, residency training combines clinical rotations with university-based theoretical teaching modules throughout postgraduate training.

Participants were categorized according to broad discipline (medicine or surgery), current semester of training and type of hospital (university versus non-university).

No formal sample size calculation was performed, as this study was designed as an exploratory national survey. Approximately 300 responses were initially expected based on the national dissemination strategy through residents’ associations, professional networks and social media channels.

### Data analysis and process of analysis

Data were processed anonymously using the Eval&GO^®^ platform. Qualitative variables are presented as counts and percentages (*n*, %) and quantitative variables as medians with IQR.

Descriptive statistics were performed using Microsoft Excel^®^ and JAMOVI^®^ (The Jamovi Project, 2024). Normality was assessed using the Shapiro–Wilk test and histogram inspection. Statistical analyses were selected according to data distribution and variable type.

For quantitative variables, the following tests were used:

- Mann–Whitney *U*-test for unpaired (independent) samples;
- Wilcoxon signed-rank test for paired samples;
- Spearman rank correlation coefficient;
- Friedman test for paired data, followed by pairwise *post hoc* analysis using the Durbin–Conover procedure;
- Kruskal–Wallis test for unpaired data with three modalities (non-parametric one-way ANOVA), followed by pairwise *post hoc* analysis using the Dwass–Steel–Critchlow–Fligner procedure.

For qualitative variables, the following tests were used:

- McNemar’s test
- χ^2^ test

Cohen’s kappa coefficient was used to assess agreement between dichotomized categorical variables beyond chance. In this context, it was used to evaluate concordance between confidence categories across related clinical situations. Interpretation was as follows: *κ* = 0 indicates agreement due to chance; *κ* ≈ 1 indicates strong agreement; *κ* < 0 indicates agreement less than expected by chance.

All statistical analyses were validated by a pharmacist with methodological expertise and were performed using JAMOVI^®^ (The Jamovi Project, 2024).

Declarative confidence responses were measured on a 0–10 numeric scale. A threshold of  ≥ 7/10 was conventionally defined as a ‘good confidence level’, encompassing high and very high ratings. For comparative analyses, scores were dichotomized into scores ≥7/10 (considered as ‘good scores’) versus <7/10 (moderate or low).

### Compliance with ethical standards

This study did not involve patients and did not include any intervention. Data were collected from anonymous medical residents through a self-administered online questionnaire and did not concern patient information.

A declaration of compliance was submitted to the French National Data Protection Authority (CNIL) in accordance with reference methodology MR-004 for non-interventional research, studies and evaluations in the field of health not involving the human person. Data were collected using the Eval&GO^®^ platform, which is compliant with the General Data Protection Regulation.

## Results

### Study population

**Figure 1.**
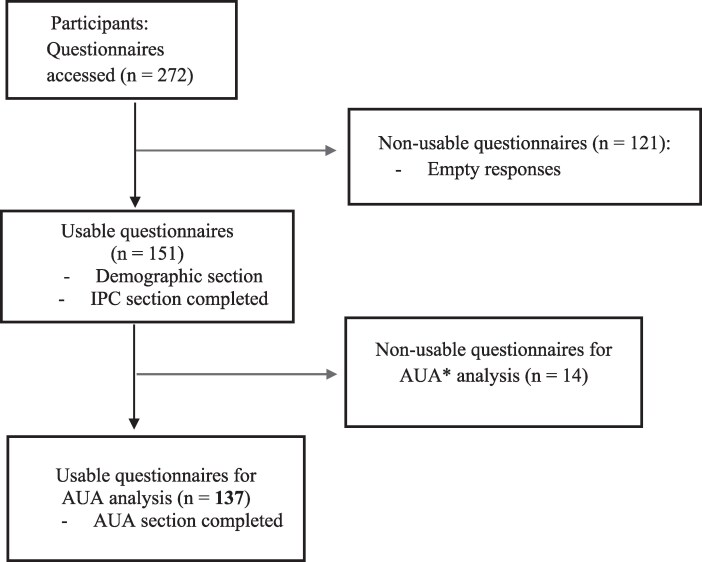
Flowchart of study participants. *AUA, appropriate use of antibiotics.

During the recruitment period, 272 individuals accessed the questionnaire and 151 residents provided analysable responses and were included in the final analysis (Figure 1).

Among respondents, 92% were medical residents (*n* = 139) and 8% surgical residents (*n* = 12). Fifty-eight per cent (*n* = 80) were in the first four semesters of residency, corresponding to the early phase of postgraduate medical training in France. Sixty-eight per cent (*n* = 103) were training at university hospitals and 32% (*n* = 48) in non-university hospitals.

### Influence of training setting

The training setting (university versus non-university hospital) was not significantly associated with residents’ LOC for IPC-related situations, including management of patients colonized with multidrug-resistant organisms (MDROs; *P* = 0.282), care of patients infected with extended-spectrum beta-lactamase–producing organisms (ESBLs; *P* = 0.136), peripheral catheter removal (*P* = 0.164) and management of blood exposure accidents (*P* = 0.162).

The training setting was significantly associated with confidence only for informing patients about MDRO colonization (*P* = 0.038), with higher median confidence among residents training in university hospitals (6.40, IQR 5.60–7.40) compared with those in non-university hospitals (5.80, IQR 4.80–6.40).

For AUA-related items, training setting was not significantly associated with confidence regarding antibiotic spectrum broadening (*P* = 0.072), antibiotic de-escalation (*P* = 0.074), management of febrile patients (*P* = 0.055), short-course antibiotic therapy (*P* = 0.611) or autonomous interpretation of antibiograms (*P* = 0.074).

Training setting was significantly associated with confidence regarding switching from intravenous to oral antibiotic therapy (*P* = 0.012), with higher median confidence among university hospital residents (7.00, IQR 6.00–8.00 versus 6.00, IQR 5.00–7.00).

### Influence of training modules

Most respondents reported having received formal training modules, including IPC modules (*n* = 105, 70%) and AUA modules (*n* = 117, 77%).

Among residents who attended IPC modules, 75% (*n* = 79) reported participation of an infectious disease specialist and 64% (*n* = 67) reported the involvement of at least one IPC practitioner.

Multidisciplinary teaching was reported by 57% (*n* = 60) of residents, two-thirds of whom were training in university hospitals.

Among residents who attended AUA modules, 99% (*n* = 100) reported the involvement of at least one infectious disease specialist.

Multidisciplinary teaching was less frequent, reported by 42% (*n* = 42), 52% (*n* = 22) of whom were university hospital residents.

Residents who reported having attended IPC training modules demonstrated significantly higher overall IPC confidence (*P* = 0.011) and higher confidence when informing patients about MDRO colonization (*P* = 0.034).

No significant association was observed between attendance of AUA training modules and confidence levels for AUA-related situations (*P* = 0.135).

### Awareness of national strategy and vaccination coverage

A large majority of residents (83%, *n* = 125) reported being unaware of the National Strategy for the Prevention of Infections and Antimicrobial resistance ((NSPIA) 2022–25).

Among the 17% (*n* = 26) who reported awareness of the NSPIA, 62% attended both IPC and AUA training modules, 42% had IPC modules involving infection control practitioners and 62% attended AUA modules involving infectious disease specialists and/or infection control practitioners.

Influenza vaccination coverage (alone or combined with COVID-19 vaccination) was 76% (*n* = 115), while COVID-19 vaccination coverage was 47% (*n* = 71).

Forty-six per cent (*n* = 69) of residents reported being vaccinated against both influenza and COVID-19, whereas 23% (*n* = 34) reported being unvaccinated against both vaccines.

Vaccination coverage differed significantly between influenza and COVID-19 vaccines (*P* < 0.001), with a higher proportion of residents vaccinated against influenza only (30%, *n* = 46) compared with COVID-19 only (1%, *n* = 2).

Vaccination status was not associated with training setting (*P* = 0.411), level of training (*P* = 0.645) or awareness of the NSPIA (*P* = 0.904).

### Comparison between AUA versus IPC confidence levels

The primary objective of this study was to compare residents’ confidence ratings across IPC- and AUA-related situations.

Median confidence scores were 6/10 for IPC-related situations and 7/10 for AUA-related situations. Overall, confidence ratings were significantly higher for AUA-related situations than for IPC-related situations (estimated difference, 95% CI 0.27–0.75, *P* < 0.001), indicating that residents reported lower confidence in IPC than in antibiotic-prescribing–related situations.

**Figure 2.**
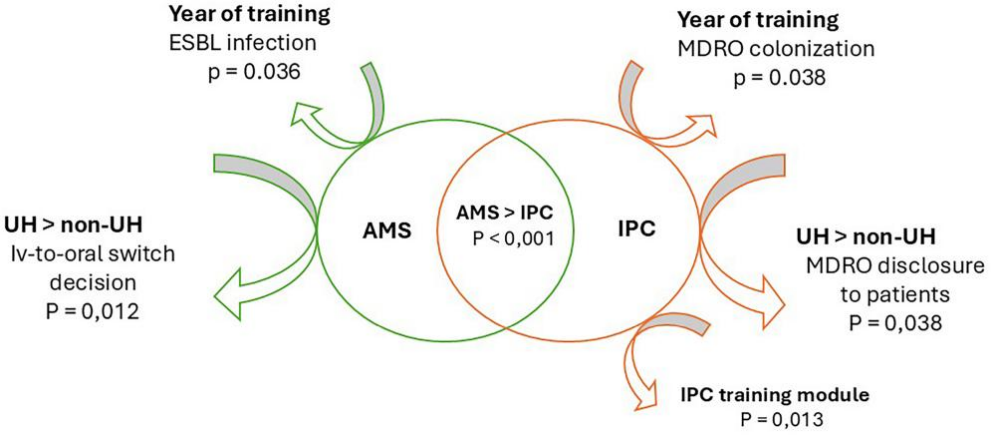
Overview of factors associated with medical residents’ confidence in AMS and IPC. UH, university hospital.

Figure 2 depicts an overview of significant factors associated with medical residents’ confidence in AMS and IPC.

### Confidence level in infection prevention and control

The IPC section was completed by 151 respondents, including 12 surgical residents and 139 medical residents. All respondents rated five IPC-related situations on a 0 to 10 numeric scale.

Residents reported significantly higher confidence when informing patients about MDRO carriage than when managing MDRO-colonized patients (*P* < 0.01). No statistically significant difference was observed between confidence in managing MDRO-colonized and ESBL-infected patients (*P* = 0.143).

Overall, years of training were not significantly associated with confidence levels, except for the management of MDRO-colonized patients (*P* = 0.038) and ESBL-infected patients (*P* = 0.036).

Analysis of dichotomized confidence scores (≥7/10) showed significant differences between good confidence in managing MDRO colonization and ESBL infection (*P* < 0.001).

Agreement between these two items was weak but greater than chance (*κ* = 0.177; *P* = 0.029), indicating heterogeneous confidence profiles across IPC situations.

Friedman testing demonstrated a significant overall difference among IPC items (*χ*^2^ = 51.3; *P* < 0.001). *Post hoc* analyses showed that residents were significantly more confident in managing MDRO-colonized and ESBL-infected patients than in informing patients, removing peripheral catheters or identifying exposure-prone situations.

### Confidence level in AUA

The AUA section was completed by 137 residents, including 11 surgical and 126 medical residents. All respondents rated six AUA-related situations on a 0–10 scale.

Residents reported higher confidence for antibiotic spectrum broadening than for de-escalation (*P* = 0.029). No significant association was observed between year of training and AUA confidence levels.

Analysis of dichotomized scores (≥7/10) showed partial concordance between AUA competencies, with weak-to-moderate kappa coefficients (*κ* ranging from 0.30 to 0.45).

These findings suggest limited concordance between confidence profiles across AUA-related tasks.

Friedman testing showed a significant difference among AUA items (χ^2^ = 67.7; *P* < 0.001). *Post hoc* analyses demonstrated that antibiotic spectrum broadening was associated with the highest confidence, compared with all other AUA situations (most *P* < 0.05).

## Discussion

This national survey assessed French medical and surgical residents’ self-reported confidence regarding IPC and appropriate antibiotic use, two key pillars of AMR prevention, which are central components of the French NAP.

Several findings merit discussion considering existing literature. First, residents reported significantly higher confidence in AUA-related situations, as a practical component of AMS, than in IPC-related situations (*P* < 0.001). In a large survey of internal medicine residents in the USA, Abbo *et al.*^6^ reported that although residents were frequently involved in antibiotic prescribing, many felt insufficiently prepared for complex stewardship decisions, yet still reported higher confidence in prescribing than in preventive or system-based measures related to infection control. This discrepancy has been repeatedly observed across healthcare systems, suggesting that routine exposure to antibiotic prescribing in daily clinical practice may foster relative confidence in AUA, whereas IPC competencies rely more heavily on procedural skills, transversal responsibilities and institutional organization, which are less directly integrated into residents’ daily decision-making. In line with this, Eriksson and Wallerstedt reported that residents’ confidence does not consistently increase with the year of training and may instead be more strongly associated with the frequency of situations encountered rather than with a strictly linear progression of competencies across semesters.^7^

Similar discrepancies between confidence in antibiotic prescribing and IPC have also been reported among medical students and early postgraduate trainees. Dyar *et al.*^8^ showed that European medical students felt more comfortable with antibiotic-related decisions than with infection prevention measures, despite acknowledging important knowledge gaps in both domains. Likewise, studies focusing on IPC education have consistently reported lower perceived preparedness among trainees, particularly regarding hand hygiene, management of MDRO carriage and exposure-prone situations. Although some of those studies involved undergraduate students rather than residents, they highlight a persistent educational gap that appears to extend into postgraduate training.

Data focusing specifically on residents are limited, but studies among internal medicine residents in Spain found that although trainees were aware of AMR as a problem, they often felt comfortable making antibiotic choices in complex scenarios (e.g. duration of therapy) despite evidence showing such decisions are challenging even for specialists.^9^ This suggests that self-reported confidence may not always reflect actual competence and that confidence in antibiotic prescribing does not necessarily correlate with optimal practice, especially when education or supervision is lacking. In this regard, our findings are consistent with those reported by Caron, who emphasized that the antibiotic susceptibility testing is not only a tool to support therapeutic decision-making but also a tool for bacterial typing, antimicrobial policy guidance and quality assessment.^10^ Importantly, the author highlighted that any positive microbiological result may act as a strong incentive to prescribe antibiotics, regardless of clinical relevance.

There is evidence that IPC education and resources vary considerably even at advanced levels of training. For example, a recent review on IPC education among infectious diseases fellows in the USA showed that many programmes lack dedicated IPC rotations and provide limited formal hours of IPC training, which could contribute to low confidence in IPC compared with clinical tasks such as prescribing.^11^

Second, we found that attendance of IPC modules was associated with higher IPC confidence. This is consistent with the literature where intervention designs that integrate case-based learning, decision support tools, audits and feedback mechanisms are reported to increase self-reported comfort with antimicrobial decisions and IPC best practices. Although this evidence comes primarily from educational programmes involving students and postgraduate trainees in mixed settings, it supports the notion that interactive, practice-oriented training may more effectively build confidence than didactic instruction alone.^12^ In contrast, a recent systematic review found that while educational interventions alone can reduce inappropriate antibiotic prescribing rates in the short term, the effect may not be durable without longitudinal or integrated system-level support.^13^

Third, the lack of a strong association between confidence and tertiary care hospital training in our study is also reflected in broader literature assessing educational resources: access to specialist training and AMS/IPC programmes may vary widely between institutions, and high-income settings do not always provide uniform access to structured stewardship or IPC education for all residents. For instance, comparative assessments of AMS and IPC activities between low/middle- and high-income countries show that formal education on these topics is inconsistent, even in resource-rich settings, suggesting that institutional setting alone is not sufficient to guarantee high confidence or competence among trainees.^14^

Fourth, the very low awareness of the national strategy for AMR among residents in our study parallels findings in other settings where trainees have limited exposure to formal institutional or national programmes on stewardship. Published evidence suggests that trainees often view AMR as a *global theoretical issue* rather than a framework that directly shapes their clinical practice. For example, in a survey of physicians in Colombia, although most acknowledged AMR as a significant global issue, only 56.9% considered it relevant to their own daily work, suggesting that AMR may be seen as more theoretical than directly impactful on clinical decision-making.^15^ Residents represent a critical target population for national AMR strategies, yet the lack of visibility of institutional policies may limit their engagement and ownership of stewardship principles. Improving awareness of national AMR strategies may require earlier and more structured integration into residency curricula, hospital induction programmes and national educational initiatives.

Vaccination coverage was higher for influenza than for COVID-19, reflecting post-pandemic vaccination fatigue and evolving risk perception among healthcare workers. Nevertheless, nearly one-quarter of residents were unvaccinated against both vaccines, highlighting persistent gaps in preventive practices even among future specialists.

This study has several limitations to acknowledge. First, its self-reported design may introduce social desirability and perception biases. Second, although the survey was disseminated nationwide and generated substantial initial participation, the final analysable sample size was lower than expected. The voluntary nature of participation may also have introduced selection bias, thereby limiting the generalizability of the findings, particularly regarding the small number of surgical residents. In addition, secondary analyses should be interpreted cautiously given the exploratory design and relatively small subgroup sizes. Third, confidence does not necessarily equate to competence, and objective assessments of knowledge or practice were not performed. Future studies combining self-reported confidence with objective knowledge assessments or clinical performance evaluations may provide a more comprehensive understanding of residents’ competencies in IPC and AMS.

Despite these limitations, this study provides valuable insights into residents’ perceived preparedness, identifies priority areas for educational interventions and paves the way for further evaluations, including pre- and post-intervention studies related to AMR.

### Conclusions

Overall, residents reported higher confidence in AUA-related situations than in IPC-related situations. This difference may reflect differences in training exposure during residency, while remaining largely independent of training in university hospital settings.

Improving residents’ engagement with national AMR strategies may further strengthen their role in combating AMR. Although IPC training was associated with higher confidence levels, current AMS education may require pedagogical adaptation to better support clinical decision-making.

Taken together, these findings support the need for earlier, multidisciplinary and practice-oriented training programmes that integrate infection prevention and AMS throughout residency, in order to contribute to curbing the projected burden of AMR by 2050.

## Supplementary Material

dlag117_Supplementary_Data

## Supplementary data

Table S1 is available as Supplementary data at *JAC-AMR* Online.
