# Supplementary material for: Confidence in antimicrobial stewardship and infection prevention among French medical residents: a national cross-sectional survey
Source: JAC Antimicrob Resist. 2026 Jun 17;8(3):dlag117. doi: 10.1093/jacamr/dlag117 (PMC13273407; doi:10.1093/jacamr/dlag117)
Supplement: dlag117_Supplementary_Data [file dlag117_supplementary_data.docx]

Supplementary Table1 : Questionnaire with its 3 sections

**Section 1**: Who am I?

➤ Residency field:

☐ Medicine

☐ Surgery

➤ Current level of training:

☐ Semester 1, 2, 3 or 4 (first two years of residency

☐ Semester 5, 6, 7 or 8 (third or fourth year of residency)

☐ Semester 9, 10, 11 or 12 (more than four years of residency)

➤ Current training institution:

☐ AP-HP

☐ AP-HM

☐ HCL

☐ Other university hospital

☐ Non-university hospital

➤ During your studies, did you receive specific training modules on infection prevention and control (IPC) ?

☐ Yes

☐ No

➤ If yes, teaching staff involved (multiple answers possible) :

☐ Infectious disease specialist

☐ Infection prevention and control practitioner

☐ Microbiologist

☐ Pharmacist

☐ Other physician

☐ Other

➤ During your studies, did you receive specific training modules on appropriate use of antibiotics and antimicrobial resistance ?

☐ Yes

☐ No

➤ If yes, teaching staff involved (multiple answers possible):
Infectious disease specialist / Infection prevention and control practitioner / Microbiologist / Pharmacist / Other physician / Other

➤ Are you familiar with the 2022–2025 National Strategy for the Prevention of Infections and Antimicrobial Resistance in human health?
Yes / No

➤ Have you been vaccinated against influenza or COVID-19 in the past 12 months?
Yes, both / Influenza only / COVID-19 only

**Section 2**: Infection Prevention and Control (IPC)

➤ On a scale from 0 to 10 (0 = not at all confident, 10 = fully confident), how would you rate your level of confidence in managing a patient colonised with a multidrug-resistant organism (MDRO)?

➤ On a scale from 0 to 10 (0 = not at all confident, 10 = fully confident), how would you rate your confidence in explaining MDRO carriage to a patient?

➤ On a scale from 0 to 10 (0 = not at all confident, 10 = fully confident), how would you rate your confidence in managing a patient infected with an extended-spectrum beta-lactamase (ESBL)–producing organism?

➤ On a scale from 0 to 10 (0 = not at all confident, 10 = fully confident), how would you rate your confidence in removing a peripheral intravenous catheter?

➤ On a scale from 0 to 10 (0 = not at all confident, 10 = fully confident), how would you rate your confidence in identifying situations at risk of healthcare-associated exposure incidents?

**Section 3:** Appropriate Use of Antibiotics (Antimicrobial Stewardship)

➤ On a scale from 0 to 10 (0 = not at all confident, 10 = fully confident), how would you rate your confidence in prescribing broad-spectrum antibiotic therapy?

➤ On a scale from 0 to 10 (0 = not at all confident, 10 = fully confident), how would you rate your confidence in de-escalating antibiotic therapy?

➤ On a scale from 0 to 10 (0 = not at all confident, 10 = fully confident), how would you rate your confidence in managing a febrile patient, whether or not antibiotic therapy is required?

➤ On a scale from 0 to 10 (0 = not at all confident, 10 = fully confident), how would you rate your confidence in prescribing a short course of antibiotic therapy?

➤ On a scale from 0 to 10 (0 = not at all confident, 10 = fully confident), how would you rate your confidence in interpreting an antibiogram independently?

➤ On a scale from 0 to 10 (0 = not at all confident, 10 = fully confident), how would you rate your confidence in switching from intravenous to oral antibiotic therapy once the patient becomes afebrile (when IV therapy was initially initiated)?
